# Mycophenolate Mofetil as a Therapeutic Option in Refractory Immunoglobulin A Vasculitis: A Report of Two Atypical Cases

**DOI:** 10.7759/cureus.85477

**Published:** 2025-06-06

**Authors:** Mohammed Al Towijry, Muhanned Amawi, Yassir M B

**Affiliations:** 1 Pediatric Department, King Salman Armed Forces Hospital, Tabuk, SAU

**Keywords:** gastrointestinal involvement, henoch-schönlein purpura (iga vasculitis), iga vasculitis (igav), mycophenolate mofetil (mmf), pediatric onset vasculitis, pulmonary hemorrhage

## Abstract

We report two rare and challenging cases of Immunoglobulin A (IgA) vasculitis with atypical clinical presentations, both of which responded favorably to treatment with corticosteroids and mycophenolate mofetil (MMF). The first case involved a patient with pulmonary-renal syndrome, a rare manifestation of IgA vasculitis. The second case presented predominantly with severe gastrointestinal manifestations and was refractory to conventional therapies, including high-dose corticosteroids, intravenous immunoglobulin (IVIG), and cryoprecipitate. In both cases, the introduction of MMF alongside corticosteroids resulted in significant clinical improvement and eventual remission. These cases underscore the potential role of MMF as an adjunctive therapy in managing severe or refractory forms of IgA vasculitis, particularly when standard treatment options prove insufficient.

## Introduction

Immunoglobulin A (IgA) vasculitis, formerly known as Henoch-Schönlein purpura, is the most common form of vasculitis in the pediatric population [1]. Its annual incidence is approximately 20.4 per 100,000 children, with the highest prevalence between four and six years of age [2]. IgA vasculitis is a small-vessel vasculitis characterized histologically by leukocytoclastic vasculitis with predominant deposition of IgA immune complexes in the vessel walls of affected organs [3].

Clinically, it is defined by the classic triad of non-thrombocytopenic palpable purpura, arthritis or arthralgia, and abdominal and renal involvement. Gastrointestinal symptoms occur in approximately 60% of patients, with around 13% requiring hospitalization for severe abdominal pain or gastrointestinal bleeding [4]. Renal involvement, observed in 30-80% of cases, ranges from microscopic hematuria and proteinuria to nephritic or nephrotic syndrome, and in severe cases, acute kidney injury [5].

Pulmonary involvement in IgA vasculitis is exceptionally rare and more frequently reported in adults. When present, diffuse alveolar hemorrhage (DAH) is the most recognized pulmonary manifestation and is often associated with concurrent renal involvement [6]. Lung involvement may initially be subclinical and should be actively investigated to prevent progression to life-threatening complications. DAH may present with symptoms ranging from mild cough and hemoptysis to severe respiratory distress and acute respiratory failure [7].

The diagnostic criteria for IgA vasculitis have evolved over time. While the 1990 American College of Rheumatology criteria were developed primarily for adults, the 2010 classification criteria established by the European League Against Rheumatism (EULAR), the Pediatric Rheumatology European Society (PReS), and the Paediatric Rheumatology International Trials Organisation (PRINTO) are now widely accepted for pediatric cases. These criteria require the presence of palpable purpura predominantly on the lower limbs, along with at least one of the following: (1) diffuse abdominal pain, (2) histopathological evidence of leukocytoclastic vasculitis or proliferative glomerulonephritis with predominant IgA deposition, (3) arthritis or arthralgia, and (4) renal involvement [8].

In this report, we present two rare and severe cases of IgA vasculitis. The first presents with concurrent pulmonary and renal involvement. The second involves gastrointestinal manifestations refractory to conventional therapies. Both patients were treated successfully with a combination of corticosteroids and mycophenolate mofetil (MMF), highlighting the potential role of MMF as an effective therapeutic option in complex or treatment-resistant cases of IgA vasculitis.

## Case presentation

Case 1

A 12-year-old previously healthy girl presented to the emergency department with a two-day history of palpable purpuric rash over the lower limbs extending to the buttocks, bilateral ankle arthritis, pedal edema, and a persistent cough. She also reported abdominal pain and vomiting during admission. Three days ago, she had experienced fever and sore throat with known exposure to siblings with upper respiratory tract infections.

**Figure 1:**
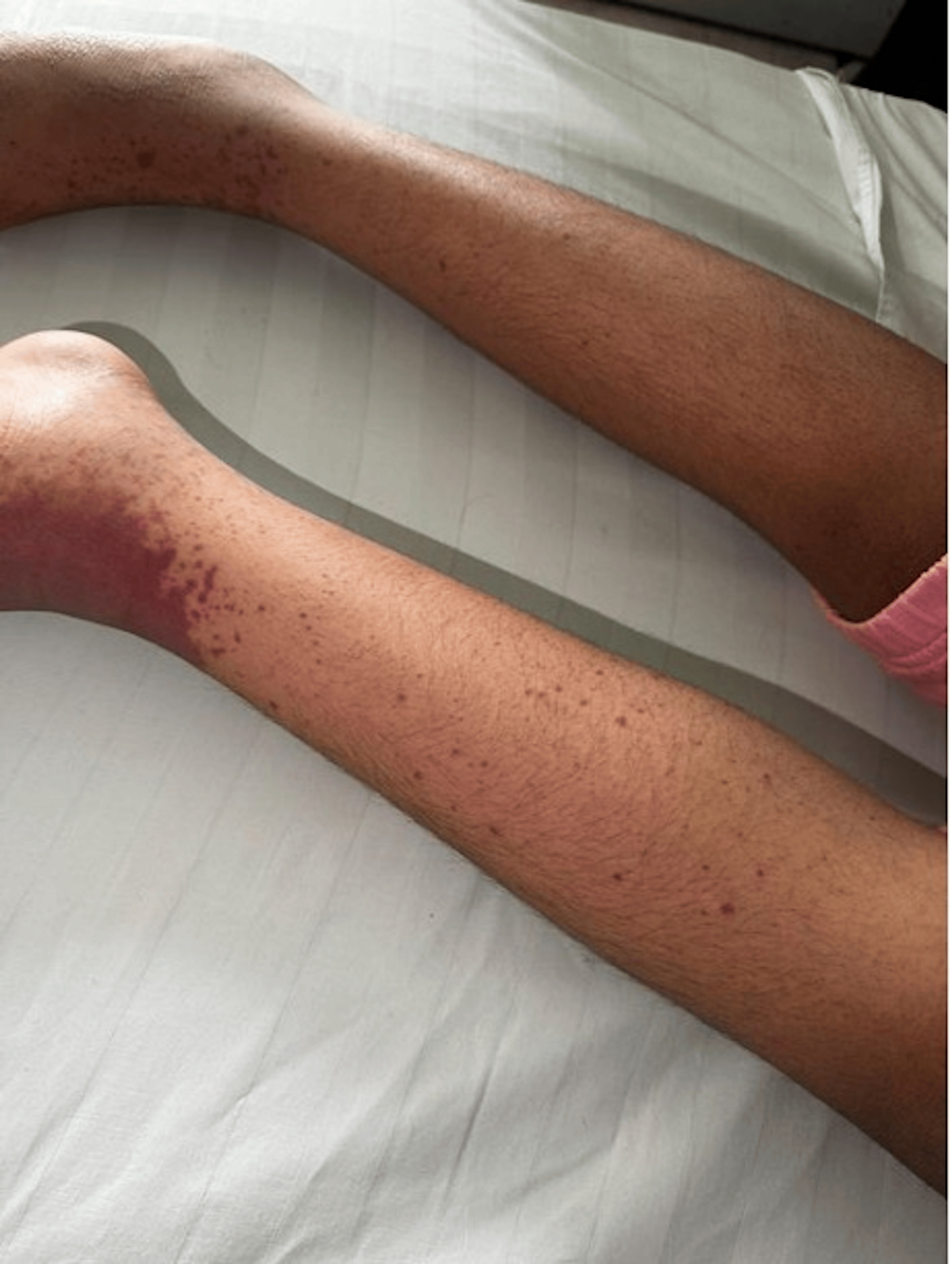
Purpuric skin rash in Case 1 patient

On examination, she was mildly dehydrated with purpuric rash, an ecchymosis over the dorsum of the left foot, bilateral foot edema, and ankle arthritis. The distribution and morphology of the rash and hematoma were documented at presentation (Figure 1). She was admitted with a clinical diagnosis of IgA vasculitis. Initial laboratory investigations revealed a normal complete blood count, liver function, and renal function. However, C-reactive protein (CRP) was elevated at 4.6 mg/dL. Urinalysis and dipstick testing were negative for proteinuria and hematuria. After admission, prednisolone was initiated at 2 mg/kg/day, resulting in partial improvement in gastrointestinal symptoms, though her cough persisted.

**Figure 2:**
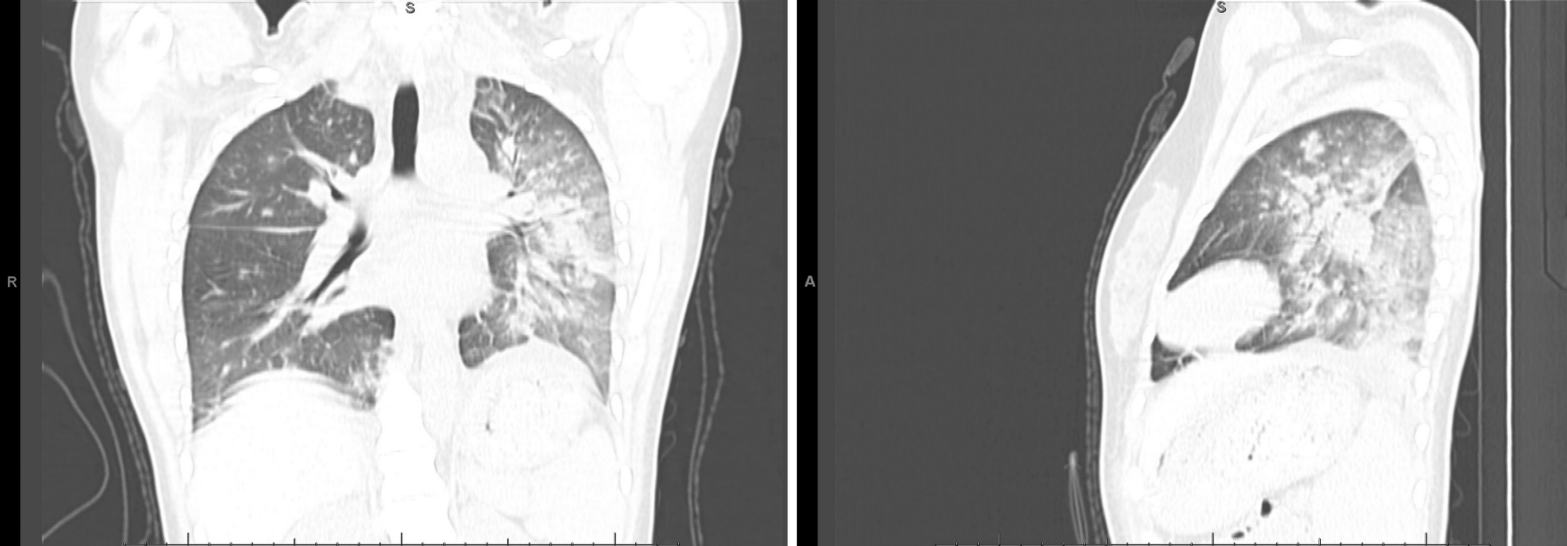
CT chest images Left-hand side image (coronal) and right-hand side image (lateral) CT chest findings for Case 1 (alveolar and nodular densities in the left lower and posterior upper lobes, scattered nodular opacities in the right lung, bilateral ground-glass changes).

By the third day of admission, she developed acute respiratory distress with oxygen saturation dropping below 90% on room air, necessitating nasal cannula oxygen, later escalating to high-flow nasal cannula at 18 L/min. Chest CT demonstrated widespread alveolar and nodular densities in the left lower and posterior upper lobes, scattered nodular opacities in the right lung, bilateral ground-glass changes, and mild pleural effusion (Figure 2). Pulmonology and radiology review concluded the findings were consistent with pulmonary hemorrhage.

At the same time, repeat laboratory testing revealed significant deterioration (Table 1). Her urine protein-to-creatinine ratio was 7.19 g/day, indicating nephrotic-range proteinuria, and urine microscopy showed 5-10 red blood cells per high-power field. Hemoglobin dropped from an initial 12.7 g/dL to 8.5 g/dL. Serologic workup revealed markedly elevated anti-streptolysin O (ASO) antibodies at 1974 IU/mL (normal <250), while complement levels were notably low: C3 was 0.2 g/L (normal 0.8-1.5) and C4 was <0.056 g/L (normal 0.12-0.36). Autoimmune screening was negative, including antinuclear antibody (ANA), Anti-double-stranded deoxyribonucleic acid antibodies (anti-dsDNA), cytoplasmic antineutrophil cytoplasmic antibody (c-ANCA), perinuclear anti-neutrophil cytoplasmic antibodies (p-ANCA), and antiphospholipid antibodies.

**Table 1:** Case 1 laboratory investigations

Parameters	Patient Values	Reference Range
Urine protein/creatinine Ratio	7.19 g/day	<0.3 g/day
Urine microscopy red blood cells per high power field (RBC/HPF)	5–10	0–3 RBCs/HPF
Hemoglobin	8.5 g/dL (from 12.7)	12–16 g/dL (female)
Anti-streptolysin O (ASO)	1974 IU/mL	<250 IU/mL
Complement C3	0.2 g/L	0.8–1.5 g/L
Complement C4	<0.056 g/L	0.12–0.36 g/L
Antinuclear antibody (ANA)	Negative	Negative
Anti-double-stranded deoxyribonucleic acid antibodies (dsDNA Abs)	Negative	Negative
Cytoplasmic antineutrophil cytoplasmic antibody (c-ANCA)	Negative	Negative
Perinuclear anti-neutrophil cytoplasmic antibodies (p-ANCA)	Negative	Negative
Antiphospholipid antibodies	Negative	Negative

Given the evidence of pulmonary hemorrhage and nephrotic-range proteinuria, and in the absence of access to bronchoalveolar lavage or renal biopsy, a multidisciplinary team (rheumatology, nephrology, pulmonology) recommended initiating pulse methylprednisolone at 30 mg/kg/day for three days along with mycophenolate mofetil (MMF). The patient responded favourably: respiratory status improved, oxygen support was gradually weaned, urinary abnormalities resolved, and systemic symptoms, including rash and gastrointestinal complaints, subsided. Steroids were tapered gradually, with MMF continued as maintenance therapy. At one-year follow-up, the patient remains clinically stable on MMF monotherapy, with hemoglobin normalized to 14 g/dL, negative urinalysis, and normalized complement levels. The team plans to continue MMF for a total of two years before tapering. 

Case 2

**Figure 3:**
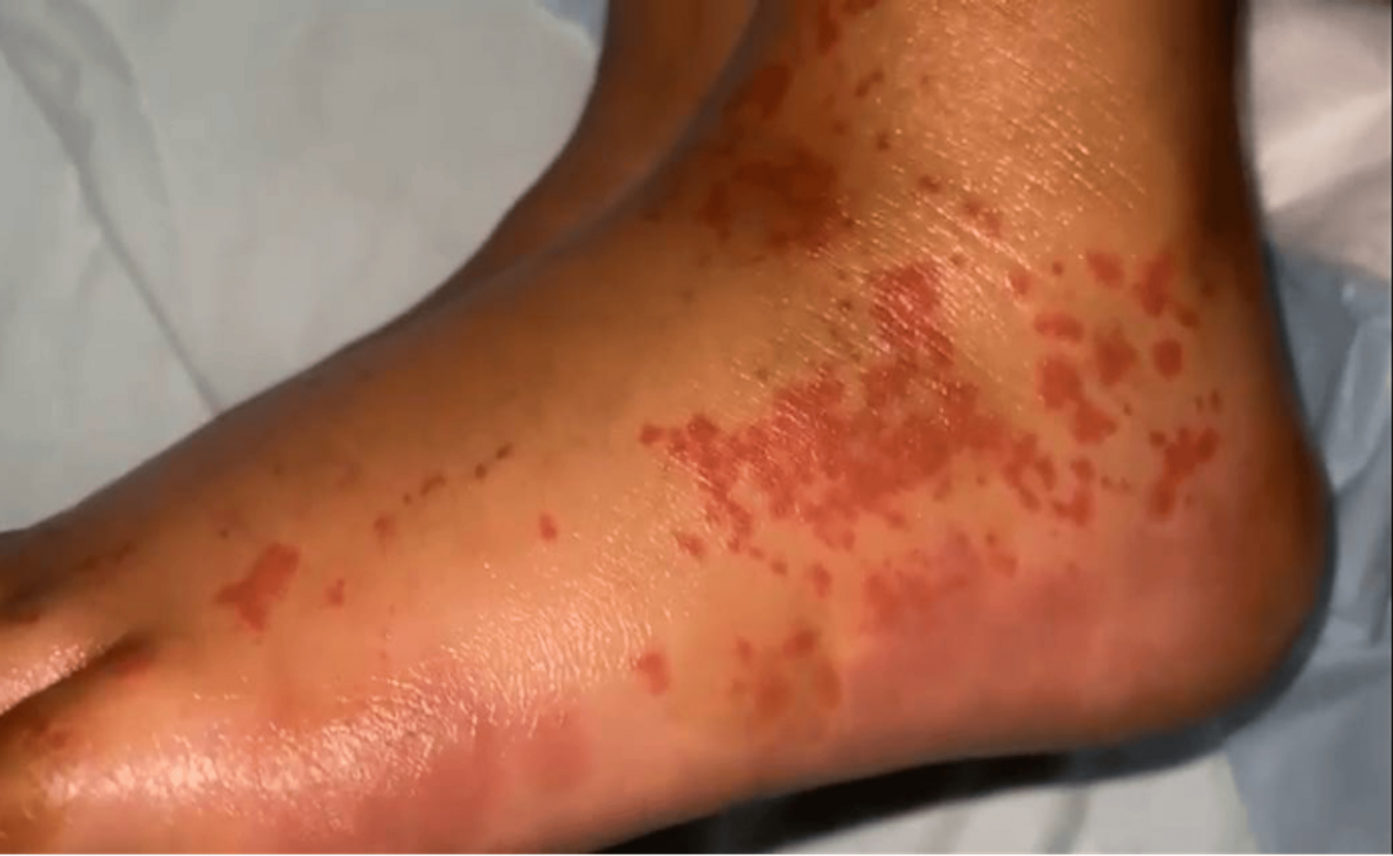
Skin rash in Case 2 patient

A seven-year-old girl, previously healthy with no chronic medical conditions, presented to the emergency department with an inability to walk and purpuric rash over the lower limbs (Figure 3). One week prior to the presentation, she had experienced an episode of acute gastroenteritis. On physical examination, there was palpable purpura on both lower limbs, along with bilateral swelling of the feet and ankles, and tenderness extending from below the knees to the dorsum of the feet.

Initial laboratory investigations, including complete blood count, erythrocyte sedimentation rate (ESR), liver function tests (LFT), renal function tests (RFT), and urinalysis, were within normal limits, except for an elevated C-reactive protein (CRP) level of 7 mg/dL (normal <1). Her symptoms improved the following day after initiation of nonsteroidal anti-inflammatory drugs (NSAIDs), and she was discharged on ibuprofen and esomeprazole.

Three days later, she was readmitted with periumbilical abdominal pain of moderate to severe intensity and reduced oral intake. There was also a reported history of blood streaks in the stool. Examination revealed recurrent purpura and lower limb edema. Repeat laboratory results were again reassuring, and abdominal ultrasound excluded intussusception. She received intravenous methylprednisolone at 10 mg/kg for three days, followed by a tapering course of oral corticosteroids at 1 mg/kg/day over one month. She was discharged on the fifth day in good clinical condition.

A third admission occurred three days after discharge due to persistent abdominal pain, refusal of oral intake, and recurrent hematochezia. As she was still on oral steroids but unable to tolerate oral intake, therapy was shifted to intravenous corticosteroids. Laboratory findings (Table 2) revealed leukocytosis (WBC 19 x10⁹/L), elevated CRP at 12 mg/dL, and ESR of 44 mm/hr (reference: 1-31 mm/hr). ANA, ANCA, and urinalysis were negative. She was administered intravenous immunoglobulin (IVIG) at 2 g/kg, which led to only partial clinical improvement.

**Table 2:** Case 2 laboratory investigations

Parameters	Patient Values	Reference Range
White Blood Cell Count (WBC)	19 x10⁹/L	4–11 x10⁹/L
C-Reactive Protein (CRP)	12 mg/dL	<1 mg/dL
Erythrocyte Sedimentation Rate (ESR)	44 mm/hr	1–31 mm/hr
(ANA)Antinuclear Antibody	Negative	Negative
(ANCA)anti-neutrophil cytoplasmic antibodies	Negative	Negative
Urinalysis	Negative	Negative
Factor XIII	55%	70%–150%

On the fourth day of admission, her symptoms persisted, including continued refusal of oral intake. Coagulation studies revealed a reduced Factor XIII level of 55% (reference: 70%-150%). Cryoprecipitate was administered at a dose of 1.5 units. Despite five days following cryoprecipitate administration, the patient remained symptomatic with ongoing abdominal pain, poor oral intake, persistent skin rash, and refusal to walk or eat. At this point, intravenous methylprednisolone was escalated to 30 mg/kg for three days, and mycophenolate mofetil (MMF) was initiated concurrently. Her condition began to improve within two days; she started eating, and the rash gradually resolved. She was discharged in stable condition after five days, with plans for outpatient follow-up while on steroids and MMF. Steroids were tapered and discontinued, and MMF was stopped after four months. During her follow-up visits, she remained asymptomatic with normal laboratory results.

## Discussion

Immunoglobulin A (IgA) vasculitis remains the most common form of vasculitis in children. Both patients described in this report fulfilled the EULAR/PRINTO/PRES criteria for the diagnosis of IgA vasculitis. Pulmonary involvement in conjunction with renal manifestations is rare in the context of IgA vasculitis. It is a life-threatening condition that necessitates prompt recognition and aggressive intervention. Most reported cases of such presentations have been documented in adults, with only a limited number described in the pediatric population. Notably, there are currently no established guidelines for managing pulmonary involvement in IgA vasculitis, and corticosteroid therapy alone does not appear to offer definitive protection [7].

Hypocomplementemia, characterized by low C3 and C4 levels, has been described as a transient phenomenon in IgA vasculitis and is often associated with elevated anti-streptolysin O (ASO) antibody titers. Importantly, no significant clinical differences or long-term sequelae have been observed between patients with and without hypocomplementemia in the context of IgA vasculitis [9]. The European consensus-based recommendations for the diagnosis and treatment of IgA vasculitis propose oral prednisolone as the first-line treatment for patients with mild renal involvement. Second-line options, including azathioprine (AZA), mycophenolate mofetil (MMF), and/or intravenous pulse methylprednisolone, are advised following renal biopsy in patients with nephritis. In our case, due to the patient’s critical clinical deterioration and the unavailability of renal biopsy services, biopsy was not pursued.

In the setting of pulmonary involvement, corticosteroids remain the initial therapeutic choice, with or without concurrent immunosuppressive agents. In adults, a combination of corticosteroids and cyclophosphamide has shown benefit. Pediatric case reports also document the use of immunosuppressive therapies, primarily azathioprine and cyclophosphamide [7]. MMF has been employed successfully for both induction and maintenance therapy in vasculitis with renal involvement. Its application in pulmonary disease has been documented primarily in connective tissue disorders. Given the potential gonadotoxicity and fertility-related concerns associated with cyclophosphamide in pediatric patients, our multidisciplinary team (rheumatology, nephrology, pulmonology) opted for MMF in combination with corticosteroids. This approach was selected based on MMF's favorable side-effect profile and its comparable clinical efficacy. The consensus was to maintain MMF therapy for two years.

Severe gastrointestinal (GI) symptoms requiring hospitalization occur in approximately 13% of IgA vasculitis cases. According to European consensus-based recommendations, such symptoms represent a valid indication for corticosteroid therapy, with oral prednisolone doses ranging from 1-2 mg/kg/day. In more severe presentations, intravenous methylprednisolone doses of 10-30 mg/kg (maximum 1 g) may be used.

Nevertheless, some patients remain refractory to corticosteroid therapy and may require second-line immunomodulatory agents. No unified guidelines currently exist for managing such steroid-resistant cases. Various therapeutic agents have been described in the literature, including intravenous immunoglobulin (IVIG), azathioprine, cryoprecipitate, cyclophosphamide, MMF, rituximab, and hemoperfusion. In a French retrospective study, intravenous immunoglobulin (IVIG) was administered in pediatric IgA vasculitis cases with severe gastrointestinal involvement unresponsive to steroids. Of eight patients, six achieved complete resolution within seven days, and two showed partial response [10]. In our case, the patient exhibited only a partial response to IVIG, with recurrence of symptoms by the third day.

Al Sonbul et al. reported three cases of IgA vasculitis that were refractory to steroid therapy [11]. One patient had also received IVIG. Cryoprecipitate was introduced after Factor XIII deficiency was confirmed in all cases, and all patients demonstrated immediate clinical improvement. The recommended transfusion dose was one unit per 10 kg body weight. However, in our case, despite administration of cryoprecipitate, the patient continued to experience abdominal pain and oral intake refusal [11]. Steroid response in IgA vasculitis with gastrointestinal manifestations may be incomplete. In such cases, introducing a second-line agent like MMF may facilitate clinical remission and allow for steroid tapering. In a cohort of 18 patients with severe abdominal symptoms and refractory IgA vasculitis, MMF administration resulted in complete remission in all cases [12].

## Conclusions

These two pediatric cases highlight the potential utility of mycophenolate mofetil (MMF) as an effective adjunctive therapy to steroids in managing refractory or severe immunoglobulin A (IgA) vasculitis. In both instances, one with pulmonary-renal syndrome and the other with persistent gastrointestinal involvement unresponsive to conventional treatment, MMF contributed to marked clinical improvement and sustained remission. Given its favorable safety profile and steroid-sparing properties, MMF may be considered a viable therapeutic option in complex cases of IgA vasculitis, particularly when standard interventions fail. Further studies are warranted to support its broader use in pediatric vasculitis care.
